# A Facile Method to Synthesize CdSe-Reduced Graphene Oxide Composite with Good Dispersion and High Nonlinear Optical Properties

**DOI:** 10.3390/nano9070957

**Published:** 2019-06-30

**Authors:** Pengchao Li, Baohua Zhu, Peng Li, Zhihao Zhang, Luyao Li, Yuzong Gu

**Affiliations:** Institute of Micro/Nano Photonic Materials and Applications, School of Physics and Electronics, Henan University, Kaifeng 475004, China

**Keywords:** CdSe/RGO nanocomposites, carboxyl groups, third-order nonlinear optical property, Z-scan technique, good dispersion

## Abstract

CdSe-reduced graphene oxide (CdSe/RGO) composites were synthesized by a hydrothermal method. CdSe/RGO composites with different mass ratios were prepared. The structure and morphology of CdSe/RGO composites were analyzed by X-ray diffraction (XRD), scanning electron microscopy (SEM) and transmission electron microscopy (TEM). The synthesis of CdSe/RGO complexes was successfully demonstrated by Fourier infrared (FT-IR) and Raman spectra. CdSe nanoparticles in the CdSe/RGO composite were uniformly dispersed on the graphene surface. The study found that oxygen-containing functional groups such as hydroxyl (-OH) and carboxyl (-COOH) groups in graphene played a decisive role in the dispersion of CdSe. The third-order nonlinear optical properties of CdSe/RGO composites were measured by a single beam Z-scan technique. The experimental results showed that composites exhibited two-photon absorption and self-focusing nonlinear refraction properties. Additionally, the third-order nonlinear susceptibility of the composite material was obviously enhanced, which was mainly due to the good dispersion of CdSe nanoparticles on graphene.

## 1. Introduction

Single-layer graphene is a thin two-dimensional material with a thickness of 0.335 nm [1], and it can exist independently with good ability [2]. Graphene is considered to be a basic building block for graphitic materials of all other dimensionalities, and it can be converted into carbon nanotubes or fullerenes by rolling and wrapping, respectively [3]. The electronic configuration is changed from the original 1s^2^2s^2^2p^2^ to 1s^2^2s^1^2p^3^ when graphite is changed to graphene. Therefore, carbon has four unpaired electrons which form the sp^2^ hybrid orbital. The s orbit and the two p orbits form a planar triangular shape around the carbon atoms [4]. The 2p orbit of each carbon atom is perpendicular to the triangular plane, so π interaction is formed between adjacent carbon atoms, meanwhile the π-band is half-filled. The special structure described above gives many excellent properties to graphene, so graphene has good electrical [5], optical [6] and other properties. In order to further broaden the application range and optimize the properties of graphene, various nanoparticles have been successfully decorated onto graphene sheets. For example, some people have already decorated SnO_2_ on graphene to form SnO_2_/graphene composite material, which exhibits an improved performance in solar cells [7]. Cheng et al. used the catalytic preparation of TiO_2_-egraphene nanocomposites to improve the catalytic properties of graphene [8]. Our group decorated graphene with CdSe and demonstrated the potential application of this composite material for nonlinear optical devices.

The discovery of a colloidal semiconductor nanoparticle has brought great significance to scientific fundamentals and technological innovation in recent years [9]. CdSe is a typical II-VI group semiconductor [10], and it has good photoelectricity and third-order optical nonlinearity etc. [11,12], which is used for bioluminescent labels [13], solar cells [14] and lasers [15]. Recently, many people successfully decorated CdSe on graphene sheets and investigated the properties of these novel nanocomposites. Chen et al. prepared CdSe/RGO nanocomposites by graphene oxide, and they found that the nanocomposite had a strong light absorption and catalytic degradation of methylene blue (MB) in aqueous solution [16]. Krishnamurthy et al. did research that discovered that GO had a greater impact on CdSe charge transfer [17]. Nyoni et al. also prepared CdSe/RGO nanocomposites and studied their electrical properties [18]. There are many people who have researched and made good progress on the properties of CdSe/RGO compounds. Yet, if the dispersion of CdSe on RGO in the composites is improved, the properties of these CdSe/RGO composites should be enhanced.

In recent years, third-order non-linearity of II-VI semiconductor and its nanocomposites with graphene have also been investigated. Recently, Zhu et al. studied the effect of synergy between CdS and graphene on nonlinear optical properties [19]. Zhu et al. researched the nonlinear optical properties of oxygen-containing defects in CdS/graphene [20,21]. Yin et al. invented a nonlinear optical imaging technique by nonlinear optical symmetry [22]. It was demonstrated that electron scattering had a critical impact on the nonlinear optical properties of graphene nanoribbons [23]. Our group also found that CdSe has good third-order nonlinear optical properties [11,12]. It can be inferred that CdSe/RGO nanocomposites should have higher third-order nonlinear optical properties than CdSe nanoparticles. The reason is that the electrons of graphene nanosheets in the composite are transferred to the conduction band of CdSe, which increases the conduction rate between electrons and enhances the nonlinear optical properties of CdSe [24]. However, as far as we know, the third-order nonlinear optical properties of CdSe/RGO nanocomposites has not been studied, so we studied their third-order nonlinear optical properties.

Herein, we have reported a facile method of decorating CdSe on reduced graphene oxide with a different concentration of CdSe nanoparticles. The morphology and structure analysis showed that CdSe nanoparticles were uniformly decorated on graphene sheets in CdSe/RGO nanocomposites. The third-order nonlinear optical properties of CdSe/RGO composites were much higher than monomeric materials, which could be attributed to the good dispersion of CdSe nanoparticles on graphene.

## 2. Materials and Methods

### 2.1. Materials

All the reagents which included graphite powder (C, ≥99.9%, Guangfu Fine Chemical Research Institute, Tianjin, China), potassium permanganate (KMnO_4_, ≥99.5%, Kermel Chemical Reagent Inc., Tianjin, China), concentrated phosphoric acid (H_3_PO_4_, ≥98.0%, Fuyu Fine Chemical Inc., Tianjin, China), concentrated sulfuric acid (H_2_SO_4_, ≥98.0%, Fuyu Fine Chemical Inc., Tianjin, China), hydrochloric acid (HCl, 36–38%, Fuyu Fine Chemical Inc., Tianjin, China), hydrogen peroxide (H_2_O_2_, ≥30%, Kermel Chemical Reagent Inc., Tianjin, China), absolute ethanol (CH_3_CH_2_OH, ≥99.7%, Fuyu Fine Chemical Inc., Tianjin, China), sodium sulfite (Na_2_SO_3_, ≥97.0%, Deen Chemical Reagent Inc., Tianjin, China), Hydrazine hydrate(N_2_H_4_·H_2_O, ≥80.0%, Fuyu Fine Chemical Inc., Tianjin, China), selenium powder (Se, ≥99.7%, Guangfu Fine Chemical Research Institute, Tianjin, China) and cadmium acetate dihydrate (Cd(CH_3_CO_2_)_2_·2H_2_O, ≥99.9%, Aladdin Biotechnology Inc., Shanghai, China) were purchased from different corporations and they were used without any further purification.

### 2.2. Synthesis of Graphene Oxide (GO)

Graphene oxide was prepared from graphite powder by Hummers’ method [25]. First, a small amount of concentrated sulfuric acid (H_2_SO_4_) and concentrated phosphoric acid (H_3_PO_4_) were mixed into a three-necked flask. Following this, the mixture of graphite powder and potassium permanganate (KMnO_4_) was slowly added into the three-necked flask. Then, the three-necked flask was heated to 50 °C and stirred for 24 h. After the reaction was completed, an appropriate amount of hydrogen peroxide solution was added to the reactants. Finally, the reaction product was ultrasonicated for 4 h, washed with hydrochloric acid and deionized water once and four times, respectively. Additionally, after removing supernatant by the centrifugal process, it was dried in a vacuum drying oven at 50 °C.

### 2.3. Synthesis of CdSe Nanoparticle

#### 2.3.1. Preparation of Sodium Selenosulfate (Na_2_SeSO_3_) Solution

First, 0.078 g selenium powder and 0.756 g sodium sulfite (Na_2_SO_3_) was added to a 150 mL three-necked flask. Then 34 mL deionized water was put in the three-necked flask. Finally, nitrogen was flushed into the three-necked flask and the three-necked flask was placed in an oil bath at 80 °C to react in an inert atmosphere. After the reaction that continued for 5 h was finished, a colorless and transparent sodium selenosulfate (Na_2_SeSO_3_) solution was obtained.

#### 2.3.2. Synthesis of CdSe Nanoparticle

First, 0.236 g cadmium acetate dihydrate and 17 mL deionized water was added to a three-necked flask. Then the sodium selenosulfate (Na_2_SeSO_3_) solution and 1.5 mL hydrazine hydrate was slowly added to the three-necked flask. The solution was mixed continuously and reacted for 3 h at 80 °C. Finally, the final product was centrifuged three times with deionized water and absolute ethanol to remove the supernatant, and then it was dried at 40 °C.

**Figure 1:**
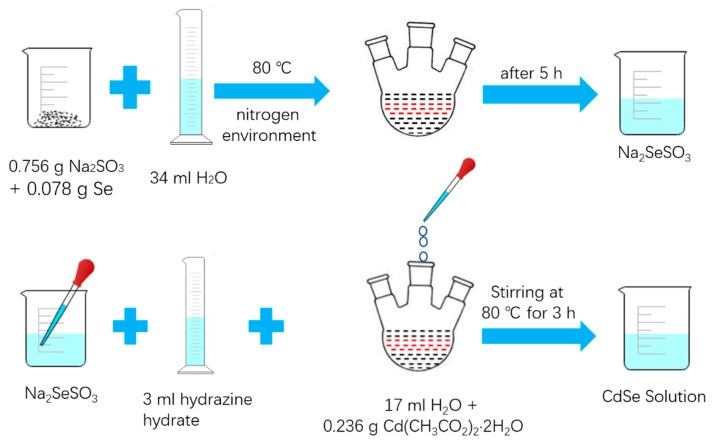
The schematic synthetic procedure of CdSe nanoparticles.

Figure 1 shows the schematic synthetic procedure of the CdSe nanoparticles. The solution gradually changed from white to light green during the reaction and then it deepened to dark green. After the reaction lasted for 10 min, the solution turned light red and gradually deepened to wine red.

### 2.4. Synthesis of CdSe/RGO Nanocomposite

The synthesis of CdSe/RGO nanocomposites was similar to the steps used in the synthesis of CdSe. First, 0.1 g graphene oxide (GO) was added to the cadmium acetate dihydrate solution, and the sodium selenosulfate (Na_2_SeSO_3_) solution and 1.5 mL hydrazine hydrate was slowly added thereafter. The mixed solution was stirred continuously and reacted for 3 h at 80 °C. Finally, it was washed three times with absolute ethanol and deionized water and dried at 40 °C. In this paper, four composites were synthesized, and the weight ratio of CdSe to GO of the complex was 1:0.5 (CdSe/RGO-1:0.5), 1:1 (CdSe/RGO-1:1), 1:1.5 (CdSe/RGO-1:1.5), and 1:2 (CdSe/RGO-1:2), respectively.

**Figure 2:**
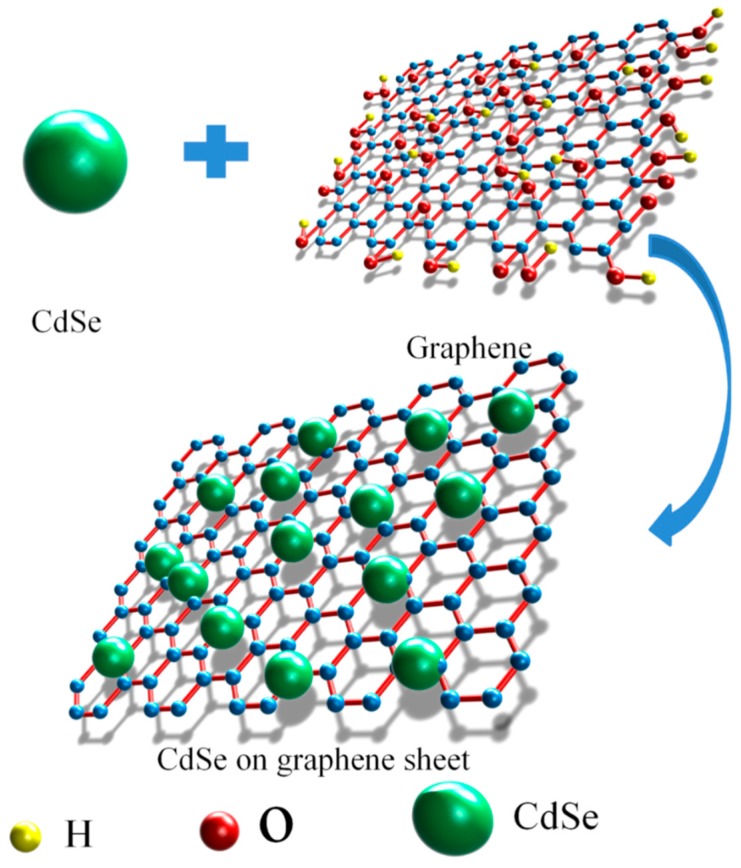
Schematic representation of the synthesis of CdSe/RGO nanocomposites.

In this paper, the CdSe nanoparticles were attached to graphene by the chemical methods. The schematic synthetic of the CdSe/RGO nanocomposite is shown in Figure 2. The CdSe nanoparticles were synthesized by following the chemical reaction process (hydrazine hydrate is strong alkalinity, it can provide an alkaline environment for the solution reaction) as shown [26]:

(1) $${\mathrm{Na}}_{2}{\mathrm{SO}}_{3}^{2-}+\mathrm{Se}\to {\mathrm{Na}}_{2}{\mathrm{SeSO}}_{3}^{2-}$$

(2) $${\mathrm{Na}}_{2}{\mathrm{SeO}}_{3}{+\mathrm{Cd}}^{2+}\to {2\mathrm{Na}}^{2+}{+\mathrm{CdSeO}}_{3}$$

(3) $${\mathrm{CdSeO}}_{3}\overset{\mathrm{slow}\;\mathrm{release}}{↔}{\mathrm{Cd}}^{2+}{+\mathrm{SeO}}_{3}^{2-}$$

(4) $${\mathrm{SeO}}_{3}^{2-}{+N}_{2}H_{4}\to {\mathrm{Se}+N}_{2}{+H}_{2}{O+2\mathrm{OH}}^{-}$$

(5) $${3\mathrm{Se}+6\mathrm{OH}}^{-}\to {2\mathrm{Se}}^{2-}{+\mathrm{SeO}}_{3}^{2-}{+3H}_{2}O$$

(6) $${\mathrm{Se}}^{2-}{+\mathrm{Cd}}^{2+}\to \mathrm{CdSe}$$

The slow-released SeO_3_^2−^ was reacted in step (4). The ultrafine Se particles, which were generated in step (4) much more easily formed Se^2−^ through the disproportioning reaction (step 5). Finally, Se^2−^ and Cd^2+^ reacted to generate CdSe nanoparticles. In our aforementioned reaction, Na_2_SeO_3_ could be more easily dissolved in the solution during the whole reaction, so we substituted Na_2_SeO_3_ for Se. This process was conducive to forming CdSe nanoparticles, which gave a high concentration. This is also beneficial for the growth of spherical CdSe nanoparticles. The moving rate and range of the CdSe monomer becomes larger during the reaction, so CdSe can form larger nanoparticles [27]. The small Ksp (solubility product constant) of CdSe drives the whole reaction and Na_2_SeO_3_ is more beneficial to the growth of CdSe nanocrystals than selenium.

When the initial CdSe nucleus formed, a large amount of N_2_ was generated in the reaction (Step 4). N_2_ can drive the CdSe monomer to form spherical CdSe nanoparticles at the same moving rate. Meanwhile, CdSe nanoparticles are attached to graphene sheets to form CdSe/RGO nanocomposites.

### 2.5. Sample Characterization

The structure and morphology of the CdSe and CdSe/RGO nanocomposite was analyzed by using field emission scanning electron microscopy (SEM, Carl Zeiss Inc., Oberkochen, Baden-Württemberg, Germany), transmission electron microscopy (TEM, JEOL JEM-2100 operating at 200 kV, JEOL Ltd. Inc., Akishima, Tokyo, Japan) and X-ray diffraction (XRD, Bruker D8 Advance, Bruker Inc., Karlsruhe, badensko-wuertembersko, Germany). The UV–vis absorption spectra were obtained by the Cary 5000 spectrometer (Agilent Inc., Sacramento, CA, USA). Raman spectra were observed on a Renishaw inVia Raman spectrometer (Renishaw Inc., Gloucester, Gloucestershire, UK) at 785 nm. Fourier transform infrared (FT-IR) spectra were measured by the Bruker Optics Vertex 70 (Bruker Inc., Karlsruhe, badensko-wuertembersko, Germany), and KBr was used as a reference for the FTIR spectrum analysis. Z-scan measurement of third-order nonlinear optical properties of samples was used. The Z-scan patterns were received by a Nd: YAG Mode-locked pulse laser (PLA2251A, Ekspla Inc., Vilnius, Lithuania) with a wavelength of 532 nm and a pulse width of 30 ps.

## 3. Results and Discussion

### 3.1. Structural and Morphology Characterization

**Figure 3:**
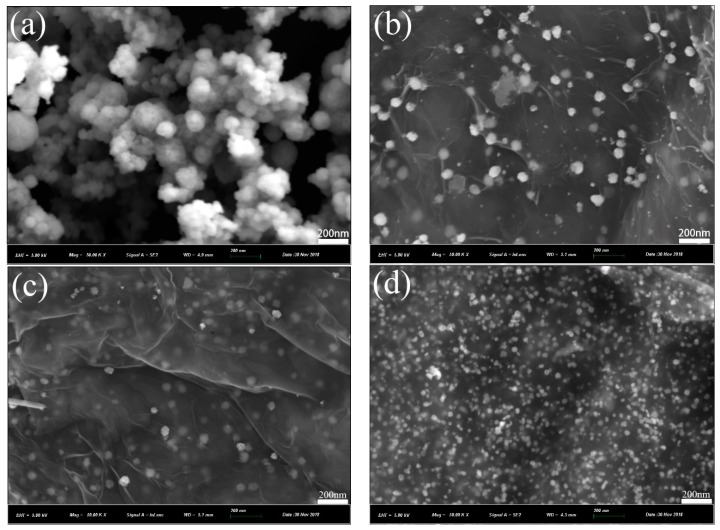
SEM images of CdSe (**a**), CdSe/RGO-1:0.5 (**b**), CdSe/RGO-1:1 (**c**), CdSe/RGO-1:1.5 (**d**).

Characterization of CdSe/RGO nanocomposites, which contain different concentrations of CdSe nanoparticles was characterized by SEM. Figure 3a is the SEM image of CdSe nanoparticles. It can be seen that CdSe are spherical particles, and CdSe nanoparticles are agglomerated together. Figure 3b–d are the SEM images of CdSe/RGO composite. It can be seen from the figure that CdSe nanoparticles in the composite gave better dispersion than pure CdSe nanoparticles. The SEM images also reveal that the amount of CdSe per unit surface area of graphene increased with no aggregate.

The reaction process (3) was the main reason for the good dispersion of CdSe nanoparticles on graphene. When CdSeO_3_ slowly released Cd^2+^, Cd^2+^ combined with the oxygen-containing functional groups (-COOH, -OH) on the graphene oxide by hydrogen bonding. The surface of the graphene sheets provided the position where the CdSe nucleus formed. Graphene can inhibit the aggregation of CdSe nanoparticles and make CdSe form smaller nanoparticles, meanwhile the CdSe prevents the stacking of graphene to a certain extent [28]. The slowly decomposed Cd^2+^ can slowly form a CdSe core at the beginning, which also plays a key role in the growth process of the core and the good crystallinity of CdSe formation.

**Figure 4:**
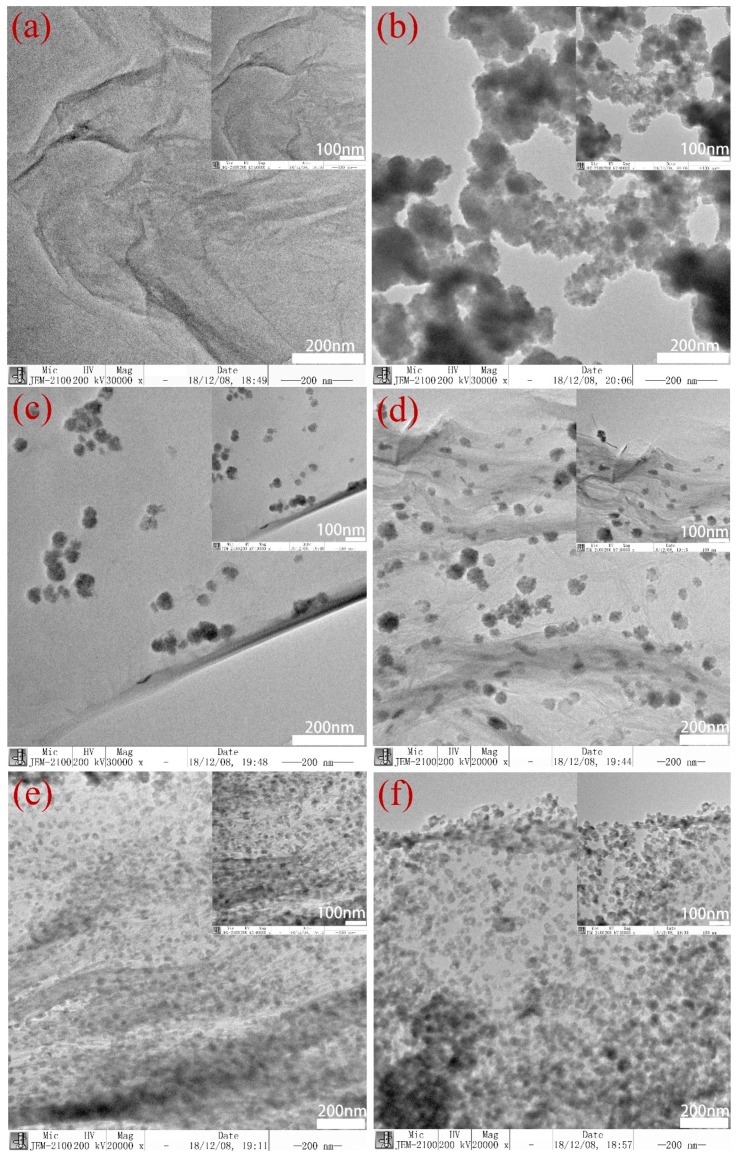
TEM images of GO (**a**), CdSe (**b**), CdSe/RGO-1:0.5 (**c**), CdSe/RGO-1:1 (**d**), CdSe/RGO-1:1.5 (**e**), CdSe/RGO-1:2 (**f**).

A Transmission electron microscope (TEM) was used to characterize the morphology of the samples. Figure 4a is the TEM image of GO. It can be seen that GO had wrinkles. Due to the appearance of these wrinkles, graphene can minimize the overall energy by adjusting the length of internal carbon bonds. Figure 4b is a TEM image of pure CdSe nanoparticle, which shows that the CdSe nanoparticles gathered together. Figure 4c–f shows TEM images of CdSe/RGO nanocomposites. We found that CdSe on graphene gradually became dense as the concentration of CdSe nanoparticles increased. Although the concentration of CdSe nanoparticles increased, CdSe nanoparticles still had good dispersion. The dispersion of CdSe was the best when the weight ratio of GO to CdSe was 1:1.5. At this point, the nanoparticles had less aggregation. As can be seen from the figure, graphene has thin layers under the CdSe nanoparticles and wrinkles. The size of the best dispersible CdSe nanoparticle was approximately 25 nm.

**Figure 5:**
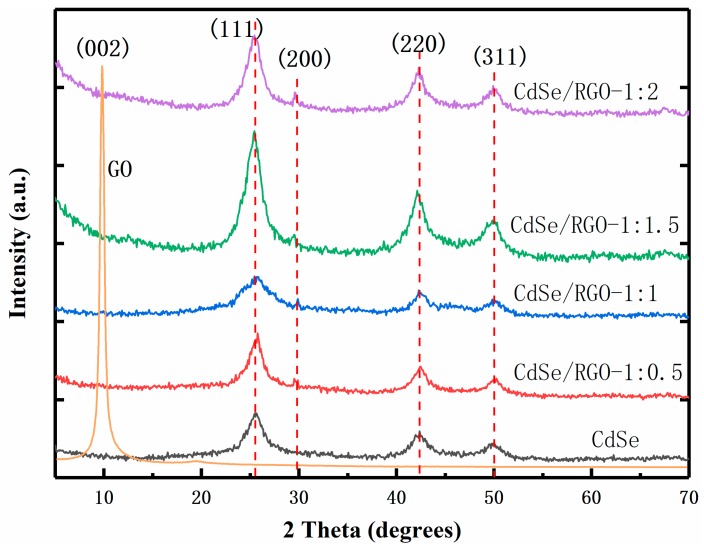
X-Ray Diffraction patterns of GO, CdSe/RGO-1:2, CdSe/RGO-1:1.5, CdSe/RGO-1:1 and CdSe/RGO-1:0.5.

The CdSe and CdSe/RGO composites were characterized by X-ray diffraction (XRD), which is shown in Figure 5. The pattern shows that graphene oxide had a single peak at 9.7°, and this diffraction peak was narrower, which indicated that the synthesized graphene oxide had good crystallinity. The diffraction peak corresponds to the (002) crystal plane of graphene oxide, which is consistent with previous reports in the literature. As for the samples of CdSe/RGO-1:2, CdSe/RGO-1:1.5, CdSe/RGO-1:1 and CdSe/RGO-1:0.5, it found that the characteristic peak of graphene oxide disappeared at 9.7 degrees when CdSe was attached to graphene. This indicates that the oxygen-containing functional groups (such as hydroxyl and carboxyl groups) on the graphene had disappeared, and it resulted in the reduction of graphene oxide. Due to the high intensity of the diffraction peak of CdSe, the diffraction peak band (2θ = 26.6°) of the reduced graphene oxide could not be observed. It can also be seen that the positions of the diffraction peaks of the composite material and CdSe were the same at 2θ = 25.5°, 29.5°, 42.2°, and 49.9°. The diffraction peaks of these four angles correspond to the (111), (200), (220), and (311) crystal planes of CdSe, respectively, which indicates that the obtained CdSe nanoparticles were a cubic zinc blende structure [11]. The diffraction pattern of the prepared CdSe nanoparticle was in good agreement with the standard diffraction pattern (JCPDS card no.65-2891). Additionally, it could be found that the highest diffraction peak of composites was composite CdSe/RGO-1:1.5, which indicated that the sample of the weight ratio has the best crystallinity.

**Figure 6:**
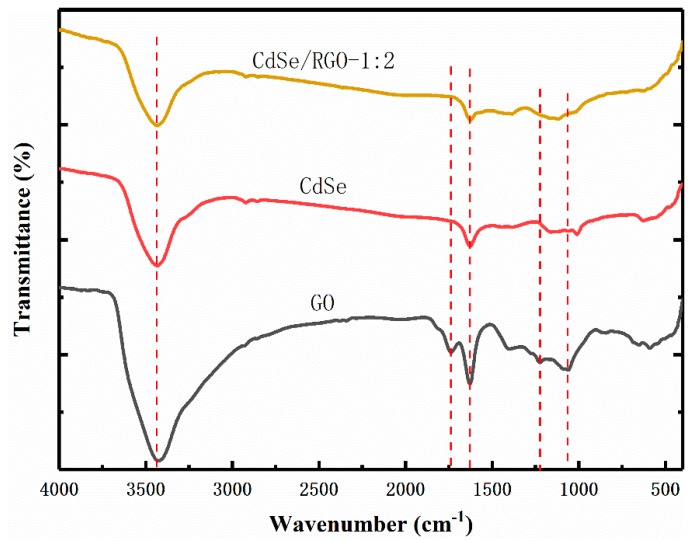
FT-IR spectra of GO, CdSe and CdSe/RGO-1:2 composite materials.

Figure 6 shows the FT-IR spectra of GO, CdSe and CdSe/RGO composite materials. For the characteristic peaks of GO, the stretching vibration peaks of O-H, C=O in –COOH, C=C, alkoxy C-O and carboxy C–OH were at 3426 cm^−1^, 1737 cm^−1^, 1629 cm^−1^, 1062 cm^−1^ and 1224 cm^−1^, respectively [29]. This suggested that the peaks of the oxygen-containing functional groups in the composite such as the hydroxyl group, carboxyl group and epoxy group were very weak. The reason might be that GO was reduced to RGO in CdSe/RGO composite material.

**Figure 7:**
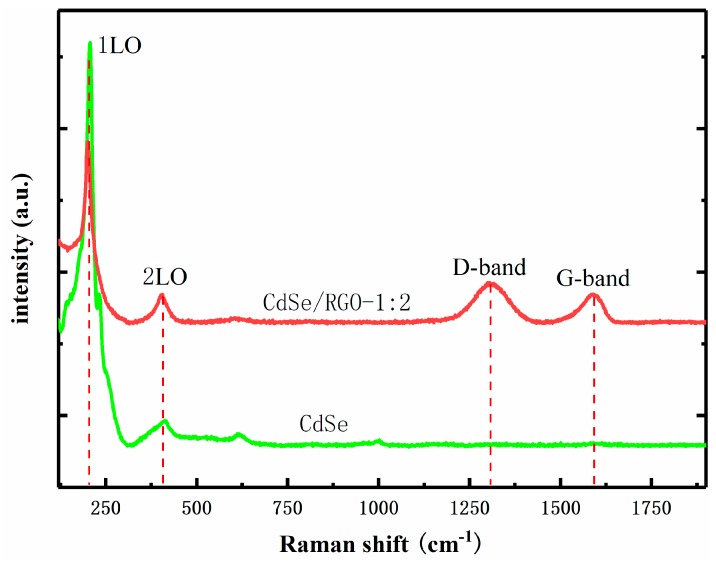
Raman spectra of CdSe and CdSe/RGO-1:2 composite materials.

Raman spectroscopy can determine the presence of CdSe and RGO in the composites. Figure 7 shows the Raman spectra of CdSe/RGO-1:2 complexes and CdSe. The Raman longitudinal optical (LO) vibration peaks of CdSe were located at 203 cm^−1^ and 409 cm^−1^, representing 1LO and 2LO peaks, respectively [30]. There were also two characteristic peaks at 1309 cm^−1^ and 1591 cm^−1^ representing the D and G bands of graphene oxide, respectively. The G-band was caused by first-order scattering of E_2_g photons. The D-band represented the graphitization degree of graphene. Therefore, the characteristic peaks of the Raman spectrum indicated that the CdSe nanoparticles grew on the RGO.

### 3.2. Linear Optical Properties

**Figure 8:**
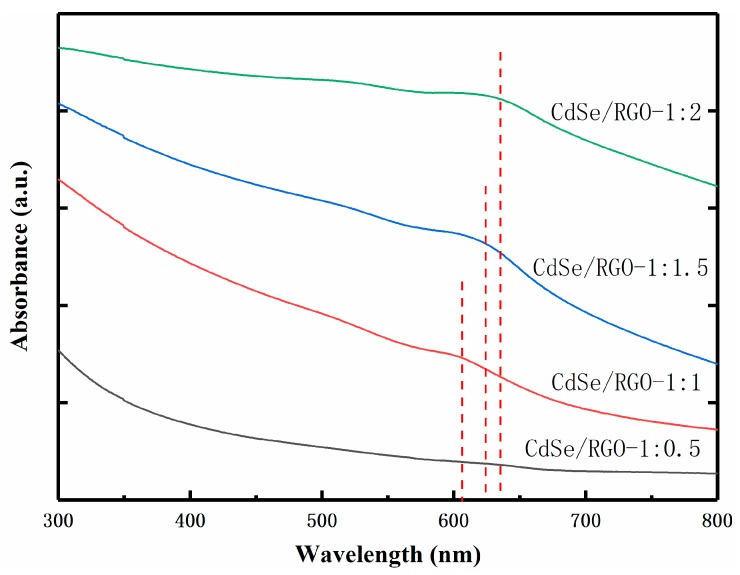
UV-Vis absorption spectra of CdSe/RGO-1:0.5, CdSe/RGO-1:1, CdSe/RGO-1:1.5 and CdSe/RGO-1:2.

Figure 8 shows the UV-Vis absorption spectra of CdSe/RGO nanocomposites. The absorption peaks positions of the complex are at 608 nm, 624 nm, and 635 nm, respectively. The absorption peak of the complex is red-shifted when the amount of CdSe nanoparticles increases. Graphene not only has high electron mobility but also suppresses recovery of electron holes. Therefore, the addition of graphene changed the electron transport mode and efficiency of CdSe nanoparticles. It is well known that graphene has no absorption peak at 400–800 nm, so the red-shifted linear absorption of composite is mainly affected by graphene.

### 3.3. Nonlinear Optical Properties

There are many methods of measuring nonlinear optical properties of nanomaterials, such as four-wave mixing, ellipsometry, and Z-scan method. We used the Z-sacn method, because it had some advantages such as the high accuracy and adequate reflection of material properties. A picosecond laser that emits a 30 ps 532 nm laser at a frequency of 10 Hz was used in the system.

**Figure 9:**
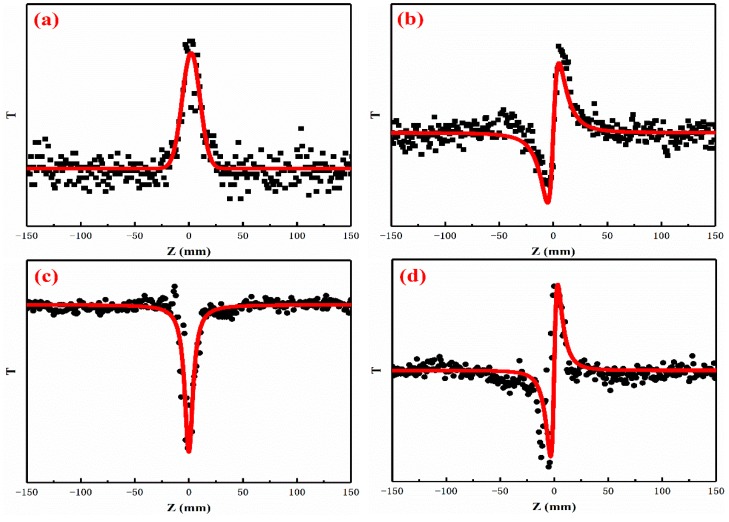
(**a**,**b**) OA and CA curve of RGO, (**c**,**d**) OA and CA curve of CdSe/RGO-1:2 composites.

Figure 9 shows the typical open-aperture (OA) and close-aperture/open-aperture (CA/OA) Z-scan curves of the RGO and CdSe/RGO nanocomposites, respectively, and the solid lines are the theoretical fitting curves. The peak and valley data plots of RGO and CdSe/RGO nanocomposites are shown in Figure 9a, c indicate that RGO had saturable absorption, and CdSe/RGO composite had two-photon absorption, respectively. The CA/OA data in Figure 9b,d show that the peak appears after valley, which indicates that RGO and CdSe/RGO composites have positive third-order nonlinear refractive index.

When composite is tested at high incident intensity, the refractive index of the medium changes with the intensity of the light, and it can be represented by [20,31]:

n = *n*_0_ + Δ*n* = *n*_0_ + *γI* = *n*_0_ + *n*_2_|*E*|^2^/2 (7)

where *n*_0_ is the linear refractive index, *γ* (*n*_2_) is the third-order nonlinear refractive index of the sample.

(8) $$n_{2}(esu)=\frac{cn_{0}}{40\pi }\gamma (m^{2}/W)$$

The theoretical curve of the normalized transmittance of the Z-scan in the actual measurement can be represented by

(9) $$T(z)=1+\frac{4x\Delta \Phi_{0}}{\left(x^{2}+9)(x^{2}+1\right)}$$

where *x* = *z*/*z*_0_, *z*_0_ is the diffraction length of the beam, Φ_0_ is the phase shift at the focus, Φ_0_(*t*) = *k*Δ*n*_0_*L_eff_*, where Δ*n*_0_ = *γI*_0_(*t*) is the nonlinear refractive index. *L_eff_* = [1 − exp(−*α*_0_*L*)] is the effective thickness of the sample, where *L* is the actual thickness of the sample and *α*_0_ is the linear absorption coefficient of the sample. The third-order nonlinear refractive index *γ* = *λ*ΔΦ_0_/2*πI*_0_*L_eff_* can be obtained by *k* = 2*π*/*λ*.

In general, nonlinear refraction is always accompanied by a certain nonlinear absorption. *α* = *α*_0_ + *βI* is absorption coefficient, where *β* is nonlinear coefficient. The normalized transmittance of the open-aperture can be expressed as:

(10) $$T(z)=\sum_{m=0}^{\infty }\frac{{\left(-q_{0}\right)}^{m}}{{\left(m+1\right)}^{3/2}}$$

where $q_{0}(z)=\frac{\beta I_{0}L_{\mathrm{eff}}}{1+{\left(z/z_{0}\right)}^{2}}$, when we meet *q*_0_ < 1, we can get:

(11) $$T(z)=\sum_{m=0}^{\infty }\frac{{\left[-q_{0}(z)\right]}^{m}}{{\left(1+m\right)}^{3/2}}\approx 1-\frac{\beta I_{0}L_{\mathrm{eff}}}{2\sqrt{2(1+z^{2}/z_{0}^{2})}}$$

After the above formula was approximated, the nonlinear absorption coefficient of the sample could be expressed as:

(12) $$\beta =\frac{2\sqrt{2}(1-T_{z=0})(1+z^{2}/z_{0}^{2})}{I_{0}L_{\mathrm{eff}}}$$

|χ(3)|=[REχ(3)]2+[Imχ(3)]2 is the third-order nonlinear susceptibility and the real and imaginary parts can be obtained through *γ* and *β* respectively, which is expressed as:

(13) $$\mathrm{RE}\chi^{\left(3\right)}=\frac{cn_{0}^{2}\gamma }{120\pi^{2}}$$

(14) Imχ(3)=λcn02β480π3

The data in Table 1 is the calculated third-order nonlinear parameters of the sample based on the above formula. It can be seen from the table that the nonlinear absorption coefficient and the third-order nonlinear susceptibility of the composite were much larger than that of the pure CdSe nanoparticles. This indicated that graphene can enhance the nonlinearity of CdSe and greatly improve the nonlinearity of nanoparticles. The main reason for the enhancement of third-order nonlinear optical properties may be due to synergistic effects and two-photon absorption. When CdSe nanoparticles are decorated onto graphene sheets, the electrons transfer rate between graphene and nanoparticles can be increased. To a certain extent, as the number of CdSe nanoparticles on graphene sheets increases, the synergy between graphene and nanoparticles becomes stronger, which makes its nonlinear properties enhanced. On the other hand, since CdSe is uniformly distributed on graphene, it nonlinearly increases with the increase of CdSe nanoparticle concentration. This is the reason why the third-order nonlinear optical properties of nanocomposite are higher than CdSe nanoparticles. This provides possibility for applying CdSe/RGO composites to nonlinear optical devices in the future.

## 4. Conclusions

In general, we synthesized CdSe nanoparticles and CdSe/RGO nanocomposites by a facile hydrothermal method. From the SEM and TEM images, we found that pure CdSe nanoparticles were particularly prone to agglomerate. When CdSe and graphene formed a composite, the nanoparticles could be uniformly dispersed on graphene sheets. This was mainly due to the effect of oxygen-containing functional groups in graphene oxide on Cd^2+^. XRD showed that the synthesized CdSe nanoparticles in the composite had a zinc-blende structure. The nonlinear absorption coefficient β and the third-order nonlinear refractive index n_2_ of the composite was found to be larger than pure CdSe nanoparticles and the maximum value reached 1.64 × 10^−10^ esu. This showed that CdSe/RGO nanocomposite is a promising material for applications in nonlinear optical devices and photoelectric switches.

## Figures and Tables

**Table 1:** The nonlinear optical parameters of the CdSe, RGO and CdSe/RGO composite materials.

Sample	Reχ^(3)^/10^−12^ esu	Imχ^(3)^/10^−12^ esu	β/cm•GW^−1^	n_2_/10^−11^ esu	χ^(3)^/10^−12^ esu
**CdSe**	3.79	7.63	11.47	2.68	8.52
**RGO**	1.12	1.42	0.75	0.21	1.81
**CdSe/RGO-1:2**	69.23	149.21	224.42	48.90	164.49
